# Return of genetic and genomic research findings: experience of a pediatric biorepository

**DOI:** 10.1186/s12920-019-0618-0

**Published:** 2019-11-27

**Authors:** Tanya Papaz, Eriskay Liston, Laura Zahavich, Dimitri J. Stavropoulos, Rebekah K. Jobling, Raymond H. Kim, Miriam Reuter, Anastasia Miron, Erwin Oechslin, Tapas Mondal, Lynn Bergin, John F. Smythe, Luis Altamirano-Diaz, Jane Lougheed, Roderick Yao, Oyediran Akinrinade, Jeroen Breckpot, Seema Mital

**Affiliations:** 1 Division of Cardiology, Labatt Family Heart Centre, Department of Pediatrics, The Hospital for Sick Children, University of Toronto, 555 University Avenue, Toronto, ON M5G 1X8 Canada; 2 Division of Clinical and Metabolic Genetics, Department of Pediatrics, The Hospital for Sick Children, Toronto, ON Canada; 3 Ted Rogers Centre for Heart Research, Cardiac Genome Clinic, Hospital for Sick Children, Toronto, ON Canada; 4 Genome Diagnostics, Pediatric Laboratory Medicine, The Hospital for Sick Children, Toronto, ON Canada; 5 Division of Medical Oncology, Department of Medicine, University of Toronto, Toronto, ON Canada; 6 Division of Cardiology, Toronto Congenital Cardiac Centre for Adults at Peter Munk Cardiac Centre, Department of Medicine, University Health Network, Toronto, ON Canada; 7 Division of Cardiology, Department of Pediatrics, McMaster Children’s Hospital, Hamilton, ON Canada; 8 Division of Cardiology, Department of Medicine, London Health Sciences Centre, London, ON Canada; 9 Division of Cardiology, Department of Pediatrics, Kingston General Hospital, Kingston, ON Canada; 10 Division of Cardiology, Department of Pediatrics, London Health Sciences Centre, London, ON Canada; 11 Division of Cardiology, Department of Pediatrics, Children’s Hospital of Eastern Ontario, Ottawa, ON Canada; 12 Program in Genetics and Genome Biology, The Hospital for Sick Children, Toronto, ON Canada; 13 Center for Human Genetics, Catholic University Leuven, Leuven, Belgium

**Keywords:** Return of research findings, Genome sequencing, Primary findings, Cost of return, Navigating return

## Abstract

**Background:**

Assess process, uptake, validity and resource needs for return of actionable research findings to biobank participants.

**Methods:**

Participants were prospectively enrolled in a multicenter biorepository of childhood onset heart disease. Clinically actionable research findings were reviewed by a Return of Research Results Committee (RRR) and returned to the physician or disclosed directly to the participant through a research genetic counselor. Action taken following receipt of this information was reviewed.

**Results:**

Genetic data was generated in 1963 of 7408 participants. Fifty-nine new findings were presented to the RRR committee; 20 (34%) were deemed reportable. Twelve were returned to the physician, of which 7 were disclosed to participants (median time to disclosure, 192 days). Seven findings were returned to the research genetic counselor; all have been disclosed (median time to disclosure, 19 days). Twelve families (86%) opted for referral to clinical genetics after disclosure of findings; 7 results have been validated, 5 results are pending. Average cost of return and disclosure per reportable finding incurred by the research program was $750 when utilizing a research genetic counselor; clinical costs associated with return were not included.

**Conclusions:**

Return of actionable research findings was faster if disclosed directly to the participant by a research genetic counselor. There was a high acceptability amongst participants for receiving the findings, for referral to clinical genetics, and for clinical validation of research findings, with all referred cases being clinically confirmed.

## Background

Return of research findings of clinical significance to participants and/or their families is a rising expectation in genetics and genomics research. This is supported by a growing body of published work on recommendations surrounding return of research findings [1–4], ethical and practical guidelines [5, 6], including guidelines specific to a pediatric population [7–9], and biobanks [10]. Most research ethics boards expect researchers to return research findings that have important health implications for the participant (and their family) and are medically “actionable i.e. have the potential to change the management of the participant and/or family” [11, 12]. The Tri-Council Policy Statement in Canada, provides guidance for the ethical conduct of research involving humans and mandates that the researcher conducting genetics research develop an ethics approved plan in their research protocol for managing information revealed during the conduct of research [13]. There are however no universally applicable guidelines that address the process of return, costs of return, resource needs, and how to deal with variability in institutional practices and regional laws [14–17]. Recommendations published by the American College of Medical Genetics (ACMG) and other working groups provide clear guidance for which secondary findings should be returned from clinical exome or genome sequencing but these do not automatically apply to return of genomic findings from research [18–20]. Several studies have explored the clinical utility of exome and genome sequencing by actively searching for variants associated with the primary disorder as well as variants in genes in the 59 reportable ACMG secondary gene list and studying the consequences of their return [21–24]. Guidelines for research studies are conflicting and the experience of navigating the return of research findings is not clear. Researchers have no ethical obligation to actively search for all actionable results [4]; however, evolving best practices place an expectation on the researcher to disclose actionable research findings if the participant was asked and consented to receive them at the time of enrollment [2]. To accomplish this, several issues need to be addressed by the researcher including consistency in identifying the clinical significance of research results, deciding which genetic results should be returned, alignment with patient or parental autonomy in choice of return of results, challenges with return of results to external participants in multi-center studies, and costs and resource needs associated with confirmation of results and the process of return.

In this study, we evaluated our early experience with return of results in a biorepository of childhood onset heart disease to determine (i) participant willingness to seek clinical genetic confirmation of disclosed findings; (ii) accuracy or validity of research findings; and (iii) efficiency of the process including time and costs of return incurred by the research team. A critical review of our experience from a disease focussed biorepository including successes and challenges in identifying, reviewing and navigating the return of findings will provide other biobanks with guidance regarding how to practically implement return of genetic and genomic research results to participants.

## Methods

### Study cohort

Study participants to whom research results were disclosed were derived from the Heart Centre Biobank Registry, a multi-center biorepository that has been prospectively enrolling pediatric and adult patients with or at risk for heart disease from six institutions across the province of Ontario, Canada since 2007 [25]. The purpose is to support clinical, genomics, and other biological research studies in childhood onset heart disease including congenital heart disease, primary and secondary cardiomyopathy, arrhythmias, and acquired heart diseases. All data and biospecimens are de-identified at the originating institution prior to transfer to the central biorepository at the Hospital for Sick Children. All participants, parents or legal guardians provided written informed consent [26]. The biorepository consent included consent for future genomics research, future re-contact, and return of research results deemed clinically “actionable”. “Actionable” findings were returned either to the primary physician or, since 2016, disclosed directly to the participant or parents through a research genetic counselor. The research was performed in accordance with the Declaration of Helsinki and the biobanking protocol was approved by institutional research ethics boards at all participating sites. This study was funded by the SickKids Labatt Family Heart Centre, the Ted Rogers Centre for Heart Research, the Heart and Stroke Foundation of Ontario Chair, the Frans Van de Werf fund for Clinical Cardiovascular Research, and the Bitove Family Professorship of Adult Congenital Heart Disease.

### Consent for return of research results

Participants were notified at the time of consent that results arising from research carried out with biobank samples including genome sequencing, would be published jointly and not returned individually. Individual results would only be returned where they were expected to provide important predictions about their or their family’s health and was likely to change management if confirmed. Language in the consent stated that the findings will be returned to their physician who would then notify them directly. After 2016, language in the research protocol and consent was amended to allow the disclosure of findings directly to the participant through a dedicated genetic counselor who was a member of the research team. The language relevant to return of results is described in Additional file 1. The genetic counselor assigned was a member of the Cardiac Genome Clinic, a funded program at the Hospital for Sick Children with experience in the process of return of genomic findings in the context of clinical and research testing [27, 28]. For families that indicated a desire to not receive research findings at the time of enrollment, the restriction was documented in the research records. No research findings were placed into the participant’s medical record.

### Reporting of research findings to the biobank

Researchers conducting research using biospecimens derived from consented participants in the Biobank were expected to return all research data back to the Biobank after study completion as part of a data and material transfer agreement. Use of biospecimens was restricted to study of the disease in question. An active search for secondary findings unrelated to the primary disorder was not expected. However, any potentially actionable findings identified during the conduct of the research project that were returned to the biobank were re-interpreted by the biobank bioinformatics team to confirm pathogenicity prior to reporting to the Return of Research Results (RRR) committee.

### Committee for Return of research results

A RRR committee was established in 2012 to develop guidelines for return and was comprised of a clinical geneticist, genetic counselor, molecular genetics laboratory director, cardiologists with expertise in cardiomyopathy and electrophysiology, research coordinator/s and bioinformaticians. This multidisciplinary team provided a rigorous assessment of clinical actionability of a variant with important health implications for the participant and/or family. The committee adjudicated each finding individually in the context of available clinical and family history. Findings concordant with available clinical phenotype were deemed reportable if they met the following criteria: (i) the finding was a sequence or copy number variant classified as pathogenic, or likely pathogenic using ACMG criteria [29, 30].; (ii) the finding was a likely cause of the primary cardiac condition, or represented a secondary finding in one or more of the 59 genes deemed reportable by the ACMG but only for those associated with a childhood onset disorder [19]; (iii) the results were not previously known to the participant on ascertainment of available medical records; and (iv) confirmatory clinical genetic testing for the research finding was available through a clinically accredited testing laboratory [18, 19]. Secondary findings associated with the risk of an adult onset disorder, secondary findings not part of the ACMG gene list, findings of uncertain significance, and negative findings were not returned. At the inception of the committee, only research findings for which targeted single gene or gene panel testing was available in a clinically accredited laboratory were included for return. With increase in research exome and genome sequencing, the guidelines were expanded to include any genetic findings that were deemed a likely cause of the primary condition or a secondary finding meeting pre-defined criteria.

### Process of return and disclosure of research results

**Fig. 1:**
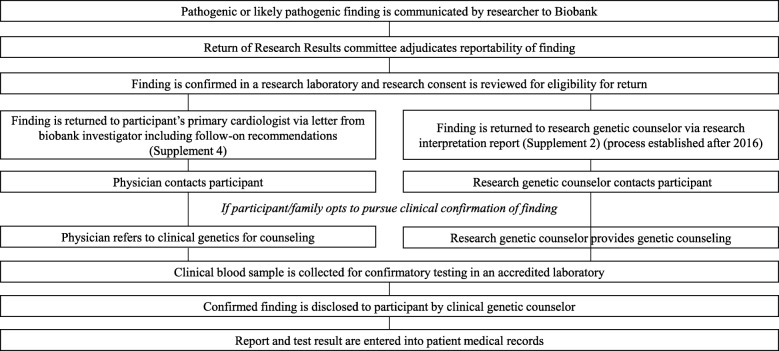
Outline of process of return of research findings

All findings that were new i.e. not previously known in the participant, and deemed reportable by the committee were first confirmed as true positive through Sanger sequencing, genotyping, or quantitative polymerase chain reaction (qPCR) in a research laboratory using a banked research DNA sample. This was performed to reduce false-positives on later clinical testing especially for complex variants identified on next-generation sequencing. Verification of participant consent for return of results and any preferences provided at the time of consent were verified prior to disclosure. Confirmed research findings for patients from our center were returned in the form of a research interpretation report generated by biobank staff to the research genetic counselor for disclosure to the participant or family (Additional file 2). The research genetic counselor (after obtaining permission from the participant’s cardiologist) disclosed to participants/families that a research finding of potential significance had been identified (by phone, mail, or in person) and offered counseling and referral to clinical genetic services for confirmatory testing in a clinically accredited laboratory after drawing a new blood sample (or using a sample available in the institutional DNA resource center) (Additional file 3). Confirmatory retesting in a clinical laboratory using a new sample is essential to avoid the potential risk of a de-identified sample mix-up in a research laboratory. The decision of the family was communicated to the physician via a letter (Additional file 4). If a family declined referral to clinical genetics for confirmatory testing, no further action was taken. If the family agreed, findings from the clinical genetic test were communicated by a genetic counselor and/or geneticist to the family and the clinical report was copied to the primary cardiologist and sent to health records. For participants from other centers, the finding was returned by the biobank to the participant’s physician, with a recommendation for the physician to contact the participant/family for their interest in confirmatory clinical genetic testing and genetic counseling (Additional file 5). Figure 1 outlines the process of return.

### Outcome after return and disclosure of results

We ascertained the actions taken after the research finding was returned to the primary cardiologist or to the genetic counselor for return to the participant by reviewing the participant’s medical records and by contacting the physician or genetic counselor to whom the results were returned. We compared the proportion of results that were disclosed to the participant, the reasons for non-return, the proportion that were clinically validated, the time to return and the costs of return incurred by the research program. Any finding communicated to the physician or genetic counselor for which there was no confirmation of disclosure to the participant within 6 months after the result was returned was considered not disclosed. Time to disclosure was calculated from the date the biobank returned the research finding to the physician or genetic counselor, until the date the participant and/or their family were notified. Costs incurred by the research program were estimated in US dollars and were based on costs for confirmatory research testing prior to return and the hourly wages for genetic counselor and research personnel time. Costs for time incurred by clinical staff (staff physician, clinical genetic counselor) on the RRR committee and for family counseling, and costs of clinical test confirmation were not included.

## Results

**Fig. 2:**
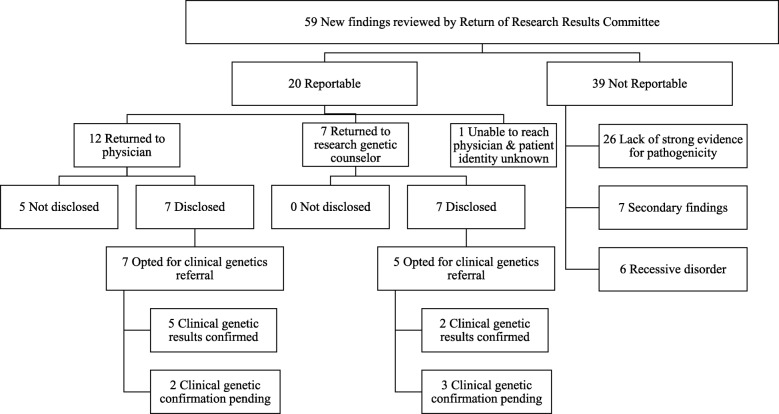
Outcome following review by the Return of Results Committee

Since the launch of the Heart Centre Biobank Registry in 2007, 7408 participants were recruited, of which 1963 have had genetic or genomic data generated as part of different research projects. The types of research tests performed included: targeted genotyping i.e. multiplex ligation probe assay for 7q11.23 deletion (*n* = 31) and 22q11.2 deletion (*n* = 10); genome-wide SNP array (*n* = 564); targeted sequencing for *ELN* (*n* = 5) and for *TBX5* (*n* = 1); exome sequencing (*n* = 619); and, genome sequencing (*n* = 733). The biobank was notified of 74 genetic results by researchers and these were presented to the RRR committee for review of which 59 results were new findings (not previously known). The decision of the committee and the outcomes associated with the return and disclosure of reportable findings are illustrated in Fig. 2.

### Adjudication on reportable findings by the RRR committee

Committee review of findings determined that 39 (66%) did not need to be returned for one of the following reasons: lack of strong evidence to support variant pathogenicity (*n* = 26), secondary finding in the ACMG gene list not associated with a childhood onset disorder (*n* = 7), or finding of a single pathogenic heterozygous variant for an autosomal recessive condition (*n* = 6). Findings deemed not reportable are detailed in Additional file 6: Table S1. 20 (34%) variants presented to the committee were deemed reportable, with potential clinical implications for the participant (e.g. finding of a variant associated with a syndrome) and/or reproductive implications for the family based on recurrence risk of the primary condition. Of these variants, 13 (65%) were in participants with congenital heart disease and 7 (35%) were in participants with cardiomyopathy. Findings are detailed in Table 1 with detailed variant information in Additional file 7: Table S2.

**Table 1:** Research findings deemed reportable by the Return of Results Committee

Type of research test	Gene: DNA change	Variant type	Zygosity	Cardiac and genetic diagnosis	Related to primary cardiac diagnosis	Research confirmation?	Returned to?	Disclosed to patient?	Accepted Referral to clinical genetics?	Clinically confirmed?
MLPA	*SNAP29-LZTR1*	Duplication	Heterozygous	PA	N	Y	Physician	Y	Y	Y
Single gene sequencing	*ELN*: c.1918 + 1G > A	Splice	Heterozygous	SVAS	Y	Y	Physician	N	N	N
Single gene sequencing	*ELN*: c.862_863insG	Frameshift	Heterozygous	SVAS	Y	Y	Physician	Y	Y	Y
Single gene sequencing	*ELN*: c.1785 T > A	Nonsense	Heterozygous	SVAS	Y	Y	Physician	Y	Y	Y
Genome-wide SNP array	Chr 1 4q34-qter deletion, 3q28ter gain	Deletion, gain	Heterozygous	BAV, AS	N	Y	Physician	Y	Y	Y
Exome sequencing	*GATA4*	Deletion	Heterozygous	AVSD	Y	Y	Physician	Y	Y	Y
Exome sequencing	*EVC*	Loss of copy	Heterozygous	AVSD	N	Y	Physician	N	N	N
Genome sequencing	*DSP*: c.C2821T	Nonsense	Heterozygous	LVNC	Y	Y	Physician	Y	Y	Result pending
Genome sequencing	*LMNA*: c.G569A	Missense	Heterozygous	DCM	Y	Y	Physician	N	N	N
Genome sequencing	*MYH7*: c.G2389A	Missense	Heterozygous	HCM	Y	Y	Physician	N	N	N
Genome sequencing	*MYBPC3*: c.G442A	Missense	Heterozygous	TOF	N	Y	Physician	N*	N	N
Genome sequencing	*PKP2*: c.337-2A > T	Splice	Heterozygous	TOF	N	Y	Physician	Y	Y	Result pending
Exome sequencing	*NR2F2*: c.A614T	Missense	Heterozygous	AVSD	Y	Y	Genetic counselor	Y	Y	Y
Genome sequencing	*FLNC*: c.3791-1G > C	Splice	Heterozygous	DCM	Y	Y	Genetic counselor	Y	Y	Result Pending
Genome sequencing	*RAF1*: c.T769C	Missense	Heterozygous	HCM	Y	Y	Genetic counselor	Y	Y	Y
Genome sequencing	*MYBPC3*: c.G3617A	Missense	Heterozygous	TOF	N	Y	Genetic counselor	Y	Y	Result Pending
Genome sequencing	*PLN*: 118795780–119,044,564	Deletion	Single copy deletion	HCM	Y	Y	Genetic counselor	Y	N	N
Genome sequencing	*VCL*: c.654dupA	Frameshift insertion	Heterozygous	TOF	Y	Y	Genetic counselor	Y	Y	Result Pending
Genome sequencing	*JAG1*: c.C514T	Stop-gain	Heterozygous	TOF	Y	Y	Genetic counselor	Y	N	N
Genome sequencing	*TPM1*: c.G688A	Missense	Heterozygous	CMP	Y	Y	Not communicated	N	N	N

*AS Aortic stenosis, AVSD Atrio-ventricular septal defect, BAV Bicuspid aortic valve, CMP Cardiomyopathy, DCM Dilated cardiomyopathy, HCM Hypertrophic cardiomyopathy, LVNC Left ventricular non-compaction, MLPA Multiplex ligation-dependent probe amplification, MR Mitral insufficiency, PA Pulmonary artery stenosis, SVAS Supravalvar aortic stenosis, TOF Tetralogy of Fallot*

*Participant was deceased and therefore result was not disclosed

### Outcome of return

Figure 2 outlines the outcome following review by the RRR. 12 of the reportable findings were returned to the physician, 7 to the research genetic counselor for disclosure to the participant, and 1 physician could not be re-contacted and the participant’s identity was not known. Of the 12 findings returned to the physician, 7 were disclosed by the physician to the participant, 5 findings were not disclosed - one because the physician mistakenly thought that the genetic diagnosis was already known, one because the patient was deceased and the reason for the other 3 is not known. In contrast, of the 7 findings returned to the research genetic counselor, all 7 have been disclosed. Therefore rate of return was 100% when disclosing results back to participants directly by the research team genetic counselor. Of the 14 results disclosed to participants, 12 families (86%) opted for clinical confirmatory genetic testing (100% when disclosed by a physician vs 71% for research genetic counselor; *p* = .039). Of the results that were sent to a clinical testing laboratory for confirmation after receiving family consent (*n* = 12), 7 were clinically confirmed, 5 results are pending. On average, the time taken by physicians to disclose the research finding to their patient was a median of 192 days (range 4–1011), while the time taken for the genetic counselor to disclose findings was shorter at 19 days (range 7–214). The one family in whom findings took longer than a month to disclose by the genetic counselor was a family that was missed during a clinic visit. Of the findings that were clinically confirmed, 3 were variants typically associated with a syndromic phenotype (*SNAP29-LZTR1*, 4q34-qter and chromosomal region gain of 3q28qter, *RAF1* pathogenic variant) but were seen in patients without syndromic manifestations and prompted additional follow-up of the patient. The method of identification, cardiac and extra-cardiac phenotypes, and follow up of all reportable variants are presented in Table 1.

### Costs of return

The costs incurred by the researcher associated with returning and disclosing research findings were divided into (i) bioinformatician and research coordinator time for manual curation of variants and review of medical records; (ii) research staff time associated with review of findings by the RRR; (iii) confirmation of finding in the research laboratory through Sanger sequencing, genotyping or qPCR; and (iv) research coordinator and genetic counselor time to return and disclose findings to participant and physician including facilitating clinical confirmation of research finding. Costs of clinical confirmatory testing were not included. Table 2 provides a breakdown of average costs per reportable finding for a total cost per reportable finding of $750 when utilizing a research genetic counselor to disclose findings directly to the participant versus $560 when returning to the participant’s physician.

**Table 2:** Average time and costs associated with return of research findings incurred by the research program

	Median time (hours)	Unit cost or hourly	Total cost
Curation of variants to present to committee			**$40**
Manual re-interpretation of variant (Bioinformatician)	0.5	$50	$25
Chart review of participant medical records (Research coordinator)	0.5	$30	$15
Return of Research Results Committee Review			
Research staff time (Bioinformatician, Coordinator, Genetic Counselor)	1	$125	**$125**
Verification of findings in research laboratory			**$425**
Design and order primers, primer optimization, PCR (Technician)	7	$30	$210
Sample retrieval (Technician)	0.5	$30	$15
Sanger sequencing (SNV), qPCR (CNV) (Technician, reagents)		$200	$200
Return of findings			**$150**
Generating research report (Research coordinator)	0.5	$30	$15
Reporting research results to patient and family counseling (Genetic counselor)	1	$45	$45
Arranging clinical validation, reporting clinical results to patient and physician, documentation (Genetic counselor)	2	$50	$100
Total research costs per reportable finding without a genetic counselor			**$560**
Total research costs per reportable finding with a genetic counselor			**$750**

## Discussion

This paper describes the practical experience of returning genomic research findings to biobank participants and how the communication process evolved to make return of results more efficient. In brief, there was a high uptake of actionable research findings by research participants as seen by a 86% opt-in for clinical genetics referral for confirmation of the research finding through clinical genetic testing. The process of return was improved by using a dedicated committee for evaluation of findings and the use of a dedicated genetic counselor in the research team for the return process, although this was associated with additional research costs.

The type of results returned by the biobank were findings associated with the primary cardiac disorder or other childhood onset disorders. Secondary findings associated with adult onset disorders were not returned due to ethical concerns regarding patient autonomy [31, 32], although some groups support returning genetic findings associated with adult onset disorders to families [18]. The majority of participants in whom results were disclosed chose to be referred to clinical genetics to pursue clinical confirmation of findings indicating that families who participate in genetics research appreciate the potential clinical value of research findings. There was a small difference in the willingness to pursue clinical confirmation by participant families between return of results by the physician (100%) versus the research genetic counselor (71%). This may reflect greater acceptance by a patient when approached by someone with whom they have a pre-existing physician-patient relationship, or it may reflect a difference in the type of information provided by a physician versus a genetic counselor.

There was a longer median time to disclosure by a physician of ~ 6 months from receiving communication from the research team. This may be related to time constraints and/or comfort level with returning genetic results [33]. Employing a genetic counselor who has the requisite expertise as a dedicated member of the research team to disclose results improved both the return rate as well as time (median time less than 1 month), and is consistent with proposed best practices [34–37]. However, the inclusion of a genetic counselor in the research team may not be feasible for all researchers and hence most researchers rely on the medical team to shoulder this responsibility. Increasing the genetic literacy of front line physicians and nurses is therefore important to improve their comfort level with interpretation of results and the process of return [23, 38]. For multi-center studies, consideration should also be given to allowing the central core institution or biobank access to patient identifiers from external sites in the event of a reportable finding so that results can be returned centrally through the biobank and minimize the burden on external sites. This can be further facilitated through use of electronic means of communication including a research portal or application [39, 40]. Providing the medical team with sample language to share with families when returning results can be helpful. In addition, an expert review panel for variant interpretation is essential to ensure high confidence in the clinical interpretation of research findings.

Costs and time required to return and disclose results are also a potential barrier to return of results [17, 41, 42]. We identified an average cost incurred by the research program of $750 per reportable research finding based on time needed by personnel for additional manual curation of results, re-interpretation of pathogenicity, additional review of medical records, committee review, counseling and research confirmatory testing. Costs incurred by a research program can be higher if a researcher performs an active search for actionable variants and performs routine re-interpretation of variants on an ongoing basis. The inclusion of a genetic counselor in the initial disclosure of genetic research findings was associated with higher costs of return to our biobank. However, it made the process of return faster and more efficient, reduced the burden of pre-test counseling on the physician, and increased the proportion of eligible results that were returned. Institutional investment at research intensive centers with large biobanks in the form of expert review panels for standardized variant interpretation, and access to genetic counseling services is important. To support this, the process of return and the costs of return should be prospectively built into the research budget of genomic projects and biobanks.

### Limitations

Since the identity of participants recruited at external sites and access to their medical records was not available to the biobank, we were unable to verify if a genetic finding was previously known prior to the return of results in some cases. Also, while we were able to confirm action taken for the participant on return of results, we were unable to verify if cascade testing in family members was performed where appropriate which would help in assessing long term health impact and healthcare costs related to return of research findings. Costs associated with return of results were reported only from the perspective of those incurred by the research program. Estimating costs associated with physician time, clinical genetic testing and clinical genetic counseling was beyond the scope of this study. Researchers accessing biobank samples were not expected to perform a systematic search for reportable variants. Therefore the yield of reportable variants in our study is likely an underestimate of all possible pathogenic findings.

## Conclusions

In summary, genetic counselor involvement as front line staff for disclosure of results is important as is increasing the genetic literacy of primary care physicians and cardiologists. Strategies to optimize the process of return to improve successful return of findings to the participants can improve participant engagement and ensure that the ethical obligations of the researcher towards participants are met.

## Supplementary information

**Additional file 1:.** Sample of Informed consent language related to return of results.

**Additional file 2:** Research interpretation report template.

**Additional file 3:** Participant contact letter template.

**Additional file 4:** Physician letter template.

**Additional file 5:** External Physician Return Letter Template.

**Additional file 6: Table S1.** Research findings deemed not reportable by the Return of Results Committee.

**Additional file 7: Table S2.** Returnable variant details.

## Data Availability

Data required to independently assess pathogenicity of all returnable variants are provided in Additional file 7: Table S2.

## Abbreviations

ACMG: American College of Medical Genetics
qPCR: Quantitative polymerase chain reaction
RRR: Return of Research Results
