# Supplementary material for: Return of genetic and genomic research findings: experience of a pediatric biorepository
Source: BMC Med Genomics. 2019 Nov 27;12:173. doi: 10.1186/s12920-019-0618-0 (PMC6882371; doi:10.1186/s12920-019-0618-0)
Supplement: Supplementary file 1 — Additional file 1:. Sample of Informed consent language related to return of results. [file 12920_2019_618_MOESM1_ESM.docx]

**Return of Results**:

In some situations, the research may yield information that may provide important predictions about the health of your child or family. In those rare situations, we will attempt to contact your physician so that they can facilitate recommended action related to research findings to you either directly or through a genetic counselor. This will only be done if there is a high likelihood of benefit to your child’s or your family’s health. This will not be possible if the results cannot be traced back to your child. The research may yield results that are of unknown significance. These results will not be returned to you. Research results will be published jointly with other study subjects. You / your child will not be identified individually when publishing these results.
