# Supplementary material for: Return of genetic and genomic research findings: experience of a pediatric biorepository
Source: BMC Med Genomics. 2019 Nov 27;12:173. doi: 10.1186/s12920-019-0618-0 (PMC6882371; doi:10.1186/s12920-019-0618-0)
Supplement: Supplementary file 2 — Additional file 2: Research interpretation report template. [file 12920_2019_618_MOESM2_ESM.docx]

**Date of Birth:**

**Sex: Female**

**Study ID:**

**Pedigree No.:**

**Ethnicity:**

**Sample Type: Blood / Saliva**

**Referring Physician:**

**Address: Heart Centre**

**CC:**

**Date Collected:**

**Date Received:**

­­­­­­­­­­­­­

| **Clinical Indications:** | Primary Diagnosis: |  |
| --- | --- | --- |
|  | Presence of known extra cardiac features?: | Y  N  Unkn |
|  | If yes, specify |  |
| **Family members tested:** | Proband Only  Proband + 1 parent  Proband + 2 Parents (trio)  Proband + sibling | |

**Results**

1. **Variants related to reported clinical phenotype**

| **Gene** | **Sequence Variant** | **Zygosity** | **Mode of Inheritance^†^** | **Interpretation** |
| --- | --- | --- | --- | --- |
|  |  | Heterozygous | AD  Parental analysis pending | Pathogenic I |

^†^ AD = Autosomal Dominant, AR = Autosomal Recessive, XL = X-linked; de novo = variant not found in parents

1. NM_022455

**2) Variants unrelated to reported clinical phenotype**

| **Gene** | **Sequence Variant** | **Zygosity** | **Mode of Inheritance^†^** | **Interpretation** |
| --- | --- | --- | --- | --- |
|  |  | Heterozygous | AD  Parental analysis pending | Pathogenic I |

^†^ AD = Autosomal Dominant, AR = Autosomal Recessive, XL = X-linked; de novo = variant not found in parents

1. NM_022455

***Interpretation:***

1. References

**Recommendations:**

It is recommended that these results be communicated to the patient in a setting that includes appropriate genetic counselling and options for confirmatory clinical testing if appropriate. Referrals for further counselling should be made if necessary.

This analysis is based on current knowledge of the molecular genetics of the clinical features indicated on the requisition. All results should be interpreted in the context of clinical findings, family history, ethnicity, other experimental data and any previous analysis of appropriate family members. Unless specifically stated it is assumed that family relationships are as indicated and that the clinical features in individuals presented as affected on the pedigree is correct.

Due to limitations of sequencing technology, a negative result does not rule out a genetic aetiology. As well, polymorphisms (benign variants), likely benign variants, variants of unknown clinical significance and secondary variants that are not medically actionable are not reported.

**RESEARCH Methodology:**

**Method:** Whole Genome Sequencing (WGS) was performed on a research basis using Illumina HiSeq proprietary sequencing technology to an average depth of at least 30X. Sequence reads were mapped to the reference genome and variants were called by local de novo assembly. Variant annotations were performed using in-house developed methods. Pathogenic variant(s) were confirmed using Sanger sequencing on a research basis.

This report has been generated as part of the XXXXXX research project and will be stored in a secure location. Only members of the research team will have access to the data.

| Choose an item.  Choose an item. | Choose an item.  Choose an item. |
| --- | --- |
