# Supplementary material for: Return of genetic and genomic research findings: experience of a pediatric biorepository
Source: BMC Med Genomics. 2019 Nov 27;12:173. doi: 10.1186/s12920-019-0618-0 (PMC6882371; doi:10.1186/s12920-019-0618-0)
Supplement: Supplementary file 3 — Additional file 3: Participant contact letter template. [file 12920_2019_618_MOESM3_ESM.docx]

Date

To the parent/guardian of

Dear Parent/Guardian,

We are contacting you regarding your child XXX’s enrollment in the Heart Centre Biobank Registry (REB# XXXX). We greatly appreciate your participation in our study and thank you for your time and participation. As we discussed during enrollment back in XXXX, part of our responsibility as researchers is to follow up with you in the event that we detect any finding during the course of your participation in the study that may be important for your child’s health or the health of your family. You indicated to us that you would like to be informed if this was the case. Through our research we have performed a test called XXXX and have identified a gene that may be associated with your child’s heart condition.

We would like to discuss these findings with you to explain their significance and a recommended course of action to confirm these findings in a clinical setting if you choose to.

Please contact us at XXXX or via email at XXXX to arrange a time to discuss further. If we do not hear from you within 2 weeks from the date this letter was sent, we will attempt to call you. If you would not like be contacted further regarding this please let us know through the contact given above.

For further information about the study, the Heart Centre Biobank Registry, please call the study main line at XXXX.

Sincerely,

XXXX (Physician) or XXXX (Genetic counselor)
