# Supplementary material for: Return of genetic and genomic research findings: experience of a pediatric biorepository
Source: BMC Med Genomics. 2019 Nov 27;12:173. doi: 10.1186/s12920-019-0618-0 (PMC6882371; doi:10.1186/s12920-019-0618-0)
Supplement: Supplementary file 4 — Additional file 4: Physician letter template. [file 12920_2019_618_MOESM4_ESM.docx]

Date: XXXX

To:

Dr. XXX

Address: XXX

Re: Return of research findings on your patient

**Participant name:**

**MRN:**

**DOB:**

**Sex:** Choose an item.

Dear Dr. XXXX,

This letter is to inform you of research findings on your patient identified through your patient’s participation in research being conducted through the Heart Centre Biobank Registry (PI: XXXX; IRB# XXXX). The findings were generated in a lab that is not an accredited or licensed clinical laboratory and are intended for research purposes only. The tests were not performed for the purposes of diagnosis, prophylaxis, or treatment.

However, analysis performed as part of the research study identified finding/s that could be of significance to the health of the participant and/or their family. These findings have NOT been verified in an accredited or licensed clinical laboratory at this time.

(*If family declines clinical confirmatory testing, use this paragraph*) Since the participant/family consented to return of research results of potential clinical significance, they were contacted by the genetic counselor of the Cardiac Genome Clinic to discuss these findings. The participant/family declined clinical confirmatory testing in an accredited or licensed clinical laboratory. Therefore, these findings will remain as research findings and no further action is required on your part.

(*If family agrees to clinical confirmatory testing, use this paragraph*) Since the participant/family consented to return of research results of potential clinical significance, they were contacted by the genetic counselor of the Cardiac Genome Clinic to discuss these findings. The participant/family decided to proceed with confirmatory testing in an accredited or licensed clinical laboratory. To facilitate this, we have included XXXX, clinical genetic counselor, on this letter so that they may assist with arranging follow-up for genetic counseling and clinical confirmatory testing through XXXX (local hospital name), and follow up on the test results. A report of the final clinical test results will be sent to you.

Please do NOT place this letter into the patient’s medical records. Should you have any questions about the research findings on your patient, please contact us at the Heart Centre Biobank Registry at XXXX.

Regards,

**RESEARCH USE ONLY: The results contained in this report were generated in a lab that is not an accredited or licensed clinical laboratory. The results are intended for research purposes only. The underlying tests were not performed for the purposes of diagnosis, prophylaxis, or treatment.**

Copy:

This letter may contain confidential and/or privileged information for the sole use of the intended recipient.  Any review or distribution by others is strictly prohibited unless explicitly specified in the text above.  If you have received this letter in error, please contact the sender and delete all copies.  Opinions, conclusions and other information expressed or contained in this letter are not given or endorsed by the sender unless otherwise affirmed independently by the sender.
