# Supplementary material for: Return of genetic and genomic research findings: experience of a pediatric biorepository
Source: BMC Med Genomics. 2019 Nov 27;12:173. doi: 10.1186/s12920-019-0618-0 (PMC6882371; doi:10.1186/s12920-019-0618-0)
Supplement: Supplementary file 5 — Additional file 5: External Physician Return Letter Template. [file 12920_2019_618_MOESM5_ESM.docx]

Date: XXXX

To:

Dr. XXX

Address: XXX

Re: Return of research findings on your patient

**Participant name:**

**MRN:**

**DOB:**

**Sex:** Choose an item.

Dear Dr.,

This letter is to inform you of research findings on your patient identified through your patient’s participation in research being conducted through the Heart Centre Biobank Registry (PI: XXXX: IRB# XXXX). The findings were generated in a lab that is not an accredited or licensed clinical laboratory and are intended for research purposes only. The tests were not performed for the purposes of diagnosis, prophylaxis, or treatment.

However, analysis performed as part of the research study identified finding/s that could be of significance to the health of the participant and/or their family. These findings have NOT been verified in an accredited or licensed clinical laboratory at this time. The patient/family consented to having research results returned to their physician in the event of discovery of clinically significant findings that could influence the child or family’s health. Please find enclosed a report detailing findings identified.

Based on these research findings the following actions are recommended, as the research team is unaware of the patient’s identity and unable to verify what clinical testing they have undergone.

1. Please verify whether this is a new finding based on their clinical history. If it is not new, no further action is required.
2. If the finding is new, please notify the patient/family that their involvement in research related to their participation in the Heart Centre Biobank Registry has identified a genetic finding related to their diagnosis of XXX. As this finding was discovered in a lab that is not an accredited or licensed clinical laboratory, these results cannot be used to influence clinical care until the finding has been verified in a clinical lab. Please only use notification as an indication warranting referral to clinical genetics should the patient be willing to pursue.
3. If the patient/family agrees to explore findings further, have the patient referred to clinical genetics for confirmation of findings.
4. Only results of clinical testing should be discussed with the patient/family. Please do not disclose findings related to research results in the event they are not clinically validated.

Please do NOT place this letter into the patient’s medical records. Should you have any questions about the research findings on your patient, please contact us at the Heart Centre Biobank Registry at XXXX.

Regards,

**RESEARCH USE ONLY: The results contained in this report were generated in a lab that is not an accredited or licensed clinical laboratory. The results are intended for research purposes only. The underlying tests were not performed for the purposes of diagnosis, prophylaxis, or treatment.**

Copy:

This letter may contain confidential and/or privileged information for the sole use of the intended recipient.  Any review or distribution by others is strictly prohibited unless explicitly specified in the text above.  If you have received this letter in error, please contact the sender and delete all copies.  Opinions, conclusions and other information expressed or contained in this letter are not given or endorsed by the sender unless otherwise affirmed independently by the sender.
